# Imipridones inhibit tumor growth and improve survival in an orthotopic liver metastasis mouse model of human uveal melanoma

**DOI:** 10.1038/s41416-024-02866-6

**Published:** 2024-10-11

**Authors:** Chandrani Chattopadhyay, Janos Roszik, Rajat Bhattacharya, Md Alauddin, Iqbal Mahmud, Sirisha Yadugiri, Mir Mustafa Ali, Fatima S. Khan, Varun Vijay Prabhu, Philip L. Lorenzi, Bo Wei, Elizabeth Burton, Rohini R. Morey, Rossana Lazcano, Michael A. Davies, Sapna P. Patel, Elizabeth A. Grimm

**Affiliations:** 1 Department of Melanoma Medical Oncology, The University of Texas MD Anderson Cancer Center, Houston, TX 77030 USA; 2 Department of Genomic Medicine, The University of Texas MD Anderson Cancer Center, Houston, TX 77030 USA; 3 Department of Surgical Oncology, The University of Texas MD Anderson Cancer Center, Houston, TX 77030 USA; 4 Department of Bioinformatics and Computational Biology, The University of Texas MD Anderson Cancer Center, Houston, TX 77030 USA; 5 Metabolomics Core Facility, The University of Texas MD Anderson Cancer Center, Houston, TX 77030 USA; 6 Chimerix, Durham, NC 27713 USA; 7 Department of Translational Molecular Pathology, The University of Texas MD Anderson Cancer Center, Houston, TX 77030 USA; 8 Department of Investigational Cancer Therapeutics, The University of Texas MD Anderson Cancer Center, Houston, TX 77030 USA

**Keywords:** Metabolomics, Melanoma

## Abstract

**Background:**

Uveal melanoma (UM) is a highly aggressive disease with very few treatment options. We previously demonstrated that mUM is characterized by high oxidative phosphorylation (OXPHOS). Here we tested the anti-tumor, signaling and metabolic effects of imipridones, which are CLPP activators, which inhibit OXPHOS indirectly and have demonstrated safety in patients.

**Methods:**

We assessed CLPP expression in UM patient samples. We tested the effects of imipridones (ONC201 and ONC212) on the growth, survival, signaling and metabolism of UM cell lines in vitro, and for therapeutic efficacy in vivo in UM liver metastasis models.

**Results:**

CLPP expression was detected in primary and mUM patient samples. ONC201 and 212 decreased OXPHOS effectors, inhibited cell growth and migration, and induced apoptosis in human UM cell lines in vitro. ONC212 inhibited OXPHOS, increased metabolic stress and apoptotic pathways, inhibited amino acid metabolism, and induced cell death-related lipids. ONC212 also decreased tumor burden and increased survival in vivo in two UM liver metastasis models.

**Conclusions:**

Imipridones are a promising strategy for further testing and development in mUM.

## Background

Uveal melanoma (UM), a rare sub-type of melanoma, is the most common primary cancer of the eye in adults and is diagnosed in about 2500 adults/year in the United States. About 50% of the UM patients progress to metastatic disease and 90% of these patients show preferential liver metastasis [1–4]. UM patients with advanced liver metastasis have poor prognosis with median survival of less than a year [2]. Metastatic uveal melanoma (mUM) does not respond to conventional chemotherapy and only rarely responds to immune therapies that are approved for cutaneous melanoma patients [5]. mUM patients currently have only two FDA approved therapeutic options: Tebentafusp (which is restricted to HLA-A*02:01-positive adult patients) for patients with unresectable disease [6] and melphalan for liver-directed therapy [7]. There remains a need for new therapeutics and strategies for mUM patients.

UM is a unique disease and distinct from cutaneous melanoma due to low mutation load and high predilection for liver metastasis (>90%) [8]. Loss of BRCA-Associated protein-1 (*BAP1*) [9, 10] and loss of a copy of chromosome 3 [11] are significant prognostic factors for UM metastasis. GNAQ and GNA11, which encode components of G-protein coupled receptors, are frequently mutated in UM [12, 13], but approved therapies to target these oncogenes are currently not available. IGF-1 pathway inhibition has shown to control UM growth in preclinical studies [14], but did not show promising outcomes in patients [15]. Thus, it is essential to identify novel vulnerabilities downstream of these aberrations to develop effective therapies.

We recently showed that UM has high OXPHOS and high expression of OXPHOS effectors like SDHA in tumors with monosomy 3 UM (M3UM), a finding independently validated by other groups [16, 17]. Unfortunately, direct OXPHOS inhibition was associated with unacceptable toxicity in patients [18, 19] and currently potent inhibitors of OXPHOS effectors are not clinically available. Imipridones are a distinct class of small molecule anti-cancer compounds [20]. Their primary mechanism of action is to activate mitochondrial protease CLPP causing dysregulated proteolysis of mitochondrial OXPHOS effectors like SDHA, SDHB, and NDUFA12, thereby reducing their cellular levels [21–23]. The imipridone compound ONC201 has shown efficacy in several preclinical models of solid tumors and hematologic malignancies, and showed promising safety, pharmacokinetic, pharmacodynamics, and efficacy in phase I/II trials involving more than 100 patients [21, 24]. ONC212, a more potent analog of ONC201, has been evaluated in multiple preclinical studies including in Acute Myelodysplastic Leukemia (AML) [25, 26]. The anti-cancer activity of imipridones demonstrated in preclinical studies, along with promising early clinical data, suggest that these compounds may present an important new agent for cancer therapy in the future. Their development will be strengthened by an improved understanding of their activity in different cancer types.

In this study, we tested ONC201 and ONC212 for their effects on UM cells in vitro and in vivo. Our studies show that in vitro treatment with ONC201 and ONC212 decreased OXPHOS effector proteins, inhibited UM cell growth, induced apoptosis, and inhibited cell migration. ONC212, which was more potent than ONC201 in vitro, robustly inhibited OXPHOS, significantly upregulated mTOR and apoptotic pathways, altered protein and redox metabolism, and increased cell death associated lipids. Importantly, ONC212 inhibited tumor growth and improved survival in vivo in orthotopic UM liver metastasis mouse models. Together, our data suggest that ONC212 may be a promising agent for clinical development in UM.

## Methods

### Antibodies and reagents

The antibodies for caspase 3 (RRID: AB_2827742; ab184787), SDHA (RRID: AB_301433; ab14715), and CLPP (RRID: AB_10975619; ab124822) were from Abcam, caspase 9 (RRID: AB_2068621), cleaved PARP (RRID: AB_10699459; 5625 s) and β1 integrin (RRID: AB_823544; ab183666) were from Cell signaling Technology, Danvers, MA, USA. β1-actin (RRID: AB_626632; sc-47778) and SDHB (RRID: AB_10659104; sc-271548) were obtained from Santa Cruz Biotechnology, Dallas, TX, USA. F-Actin was obtained from Bioss Inc., Woburn, MA, USA (RRID: AB_10859354; bs-1571R).

ONC201 and ONC212 were provided by Oncoceutics/Chimerix (Durham, NC, USA) under an MTA.

Cell migration assay plates were from Corning (354578; Corning, Glendale, AZ) and the staining kit was from Siemens Health care Diagnostic Inc., Newark, DE, USA (B4132-1A). Methylthiazole tetrazolium (MTT) reagent was from EMD Millipore Corp, Burlington, UK (#475989), Propidium iodide (81845) and ribonuclease A (RNase; R4642) from Sigma Aldrich, St. Louis, MO, USA. The Mito Stress Kit (Seahorse XF Cell Mito Stress Test Kit, #103010–100) and plates (#103794-100) were obtained from Agilent Technologies, Santa Clara, CA, USA.

### Cell culture and treatments

Cell line MEL20–06–039, (RRID: CVCL_8473) [27, 28], was obtained from Dr. Tara A. McCannel. Cell lines OMM-1 (RRID: CVCL_6939) [29], MEL202 (RRID: CVCL_C301), 92.1 (RRID: CVCL_8607) [29], and MEL270 (RRID: CVCL_C302) [29] were kindly provided by Drs. Martine Jager and Bruce Ksander. UM cell lines obtained from American Type Culture Collection (ATCC) were: MM28 (#CRL-3295), MP38 (#CRL-3296), MP41 (#CRL-3297), MP46 (#CRL-3298), MP65 (#CRL-3299). Additional UM cell line information is provided in Fig. S1A [30–33]. UM cells were cultured in RPMI 1640 media (Hyclone, #SH30096.01) with 10% FBS (Sigma-Aldrich, #12306 C), 1% glutamine (Thermo Fisher, #25030081), 1% Penicillin-streptomycin (Sigma-Aldrich, #30-002-CI), and 1% Insulin supplement (Thermo Fisher, #51300044), under ambient oxygen at 37 °C. Cell lines were validated by short random repeat (STR) DNA fingerprinting techniques and mutational analysis, by the MDACC Cancer Center Support Grant (CCSG)-supported Characterized Cell Line Core.

### Western blotting

Cells were lysed in a buffer containing 50 mM Tris (pH 7.9), 150 mM NaCl, 1% NP40, 1 mM EDTA, 10% glycerol, 1 mM sodium vanadate, and a protease inhibitor cocktail (Roche Pharmaceuticals, Nutley, NJ, USA). Proteins were separated by SDS-PAGE (Bio-Rad Laboratories, Hercules, CA, USA), transferred to a Hybond-ECL nitrocellulose membrane (GE Healthcare Biosciences, Piscataway, NJ, USA), and blocked in 5% milk. After primary and secondary antibody incubation Thermo Fisher Scientific Super Signal chemiluminescence reagent (Thermo Fisher, Waltham, MA, USA) was used for detection, and Li-COR C-DiGit Blot Scanner (LI-COR, Lincoln, NE, USA) was used for imaging and quantitation. For each marker, the western blots were standardized multiple times and appropriate representative blots are included in the figures.

### Mito stress test: seahorse analysis

The Seahorse XF Mito Stress Test was performed using a Seahorse XFe96 flux analyzer (Agilent) per the manufacturer’s protocol. Briefly, 15,000 UM cells were seeded per well of a Seahorse 96-well plate. The following day, fresh media containing vehicle, 0.1 μM, or 0.2 μM ONC212 was added, and incubated for 24 h. Prior to the assay, media was changed to Seahorse XF RPMI (Agilent, #103576-100) supplemented with 1 mM pyruvate (Agilent, #103578-100), 10 mM glucose (Agilent, #103577-100), and 2 mM glutamine (Agilent, #103579-100) for 30 mins at 37 °C in a non-CO_2_ incubator. Ports were loaded to achieve final concentrations of 2 μM oligomycin A, 0.5 μM FCCP, 1 μM rotenone/1 μM antimycin A, and 2 μM Hoechst 33342. Following the assay, nuclear staining was imaged on the Cytation 5 (BioTek, Winooski, VT, USA) and cell numbers were analyzed by XF Cell Imaging and Counting software (Agilent). Cell counts were integrated into the XF data analysis using Wave software. Oxygen consumption rates (OCRs) were normalized to cell number per well.

### Cell cycle analysis using propidium iodide staining and FACS

UM cells were trypsinized, washed, and fixed with 70% Ethanol and kept at 4 °C overnight. Cells were centrifuged and pellets were resuspended in PBS to rehydrate for 15 min. Cells were then centrifuged at 500 × *g* and treated with 200 µg/mL RNase A for 1 h at 37 °C. Cells were stained with 40 µg/mL Propidium Iodide for 20 min at room temperature. After centrifugation at 500 × *g*, cells were resuspended in PBS with 0.02% EDTA for cell cycle analysis using a Beckman Coulter Galios 561 analyzer (Beckman Coulter, Brea, CA, USA).

### Colony formation assay

For the colony formation assay, UM cells were seeded in 24 well plates at 500 cells/well, and ONC212 (0.1, 0.25 and 0.5 µM) was added the following day. Controls were untreated cells. The drug was replaced every three days. Colonies were grown until control wells reached 70–80% confluency. Cells were washed with 1X PBS and stained with 1 mL of crystal violet in 25% methanol for 5 min at room temperature. Colonies were imaged and counted. The effect of ONC212 on colony formation was quantitated by counting colonies from 10 independent fields/sample and was plotted as a bar graph.

### Reverse phase protein array (RPPA) analysis

RPPA analyses were performed at The University of Texas MD Anderson Cancer Center Functional Proteomics RPPA Core facility [14]. Briefly, cell lysates were serially diluted (from undiluted to 1:16 dilution) and arrayed on nitrocellulose-coated slides. Samples were probed with antibodies using catalyzed signal amplification and visualized by 3,3’-diaminobenzidine colorimetric reaction. Slides were scanned on a flatbed scanner to produce 16-bit TIFF images and were quantified using the MicroVigene software program (Version 3.0). Relative protein levels for each sample were determined by interpolation of each dilution curve. Heatmaps were generated in Cluster 3.0 (http://www.eisenlab.org/6isen/) (accessed on 08/11/2023) as a hierarchical cluster using Pearson correlation and a center metric. RPPA data were normalized by antibody then by sample.

For pathway analysis, RPPA data were median-centered and normalized by standard deviation across all samples for each component to obtain the relative protein level. The pathway score is then the sum of the relative protein level multiplied by its weight of all components in a particular pathway [34].

### Cell viability assays

MTT- based cell viability assays were used for estimating cell survival. UM cells were plated at a density of 1 × 10^4^ cells/well in triplicate in a 24-well plate. Next day, ONC201 and/or ONC212 was added to the cells in designated doses and incubated for 72 h. To assess cell viability, MTT reagent dissolved in PBS was added to wells for a final concentration of 1 mg/mL. After 2 h the precipitate formed was dissolved in DMSO, and the color intensity was estimated in an MRX Revelation microplate absorbance reader (Dynex Technologies, Chantilly, VA, USA) at 570 nm.

### Cell migration assay

Cell migration assays were performed in Boyden chambers using uncoated filters (BD Biocoat control inserts). UM cells were plated and treated overnight with ONC212 (0.2 µM). Untreated cells were used as a control. The next day, 1 × 10^5^ cells/well were plated in a serum-free medium, and the migration assay was completed with 10% FBS as a chemo-attractant and procedure as described in Chattopadhyay et al. [35]. Stained cells were photographed with a Nikon Eclipse TE2000-U microscope (Nikon Instruments Inc., Tokyo, Japan) at ×20 magnification using Nikon’s NIS Elements advanced research software. To quantify migration, the cells in each filter were counted from five independent fields under the microscope at ×40 magnification and the mean cell number/field was calculated. Each assay condition was tested in duplicates.

### Metabolomics profiling

Metabolites were extracted using 1 mL of ice-cold 0.1% ammonium hydroxide in 80/20 (v/v) methanol/water. Extracts were centrifuged at 17,000 × *g* for 5 min at 4 °C, the evaporated supernatants were reconstituted in deionized water, and 10 μL were injected for analysis by ion chromatography—mass spectrometry (IC-MS). Data were acquired using a Thermo Orbitrap Fusion Tribrid Mass Spectrometer under ESI negative ionization mode at a resolution of 240,000. Raw data files were imported to Thermo Trace Finder software for final analysis. The relative abundance of each metabolite was normalized by total area, log transformed, and scaled by z-score. Metabolite data were processed and annotated using Thermo Fisher Scientific Compound discoverer software (version 3.3 SP2) and relative abundance and metabolic pathway analyzed using R scripts written in house.

### Lipidomics profiling

To each cell sample, 200 µL of extraction solution containing 2% Avanti SPLASH® LIPIDOMIX® Mass Spec Standard, 1% 10 mM butylated hydroxytoluene in ethanol was added and vortexed 10 min, incubated on ice for 10 min and centrifuged at 13,300 rpm for 10 min at 4 °C. 10 µL of supernatant was injected. Mobile phase A (MPA) was 40:60 acetonitrile: water with 0.1% formic acid and 10 mM ammonium formate. Mobile phase B (MPB) was 90:9:1 isopropanol: acetonitrile: water with 0.1% formic acid and 10 mM ammonium formate. The chromatographic method included a Thermo Fisher Scientific Accucore C30 column (2.6 µm, 150 × 2.1 mm) maintained at 40 °C, autosampler tray chilling at 8 °C, a mobile phase flow rate of 0.200 mL/min, and a gradient elution program as follows: 0–3 min, 30% MPB; 3–13 min, 30–43% MPB; 13.1–33 min, 50–70% MPB; 48–55 min, 99% MPB; 55.1–60 min, 30% MPB. A Thermo Fisher Scientific Orbitrap Fusion Lumos Tribrid mass spectrometer was operated in data dependent acquisition mode, in both positive and negative ionization modes, with scan ranges of 150–827 and 825–1500 m/z. An Orbitrap resolution of 120,000 (FWHM) was used for MS1 acquisition and a spray voltage of 3600 and –2900 V were used for positive and negative ionization modes, respectively. Vaporizer and ion transfer tube temperatures were set at 275 and 300 °C, respectively. The sheath, auxiliary and sweep gas pressures were 35, 10, and 0 (arbitrary units), respectively. For MS2 and MS3 fragmentation a hybridized HCD/CID approach was used. Lipid data were processed and annotated using Thermo Fisher Scientific LipidSearch software (version 5.0) and analyzed using R scripts written in house.

### Tumor models for UM metastasis

#### Generation of luciferase tagged UM vells

The four UM models that were used for generating orthotopic liver growth of UM to determine the effect of ONC212 on tumor growth were tagged with luciferase using lentiviral infections. The lentivirus was purchased from GenTarget Inc. (LVP020-PBS).

#### Splenic injection

We followed the splenic injection methodology as described in Sugase et al. [36]. Briefly, 6-8 wk old NSG mice (RRID: IMSR_JAX:005557; from Jackson Laboratory, Bar Harbor, ME, USA) were placed in the right lateral recumbent position. A 1 cm incision was made in the left upper abdominal wall, followed by a 1 cm incision in the peritoneum to expose the spleen. 0.5 × 10^6^ cells in 50 μL of HBSS were gently injected into the spleen. The insertion site of the needle was cauterized and sealed with sutures to curtail bleeding. Splenectomy was performed 15 min after injection using surgical cautery tip (#231; McKesson, Irving, TX, USA). The abdominal incision was closed in two layers with 5-0 polydioxanone absorbable thread (AD Surgical, Sunnyvale, CA, USA). Mice were injected with Buprenorphine SR subcutaneously for pain relief (1 mg/kg) and kept under observation for 72 h.

#### Monitoring tumor growth via in vivo imaging

For luciferin-based imaging, mice were anesthetized by the XGI-8 Gas Anesthesia system (2% isoflurane; Xenogen, Alameda, CA, USA) [37]. After 10 min of intraperitoneal injection of luciferin (150 mg/kg body weight), bioluminescence intensity was measured by In Vivo Imaging System (IVIS)-200 (Xenogen). Image sequence was acquired using Living Image (Xenogen) software. The images were cropped to fit the final figures using Adobe Photoshop Software.

#### ONC212 treatment

After confirming tumor growth in the liver, mice were randomized by quantitation of bioluminescence from the IVIS scans for subsequent treatment with ONC212. To reduce variability due to confounding factors, mice were maintained on a standard diet and all mice in the study were co-housed. Mice from the same litter were used for the study and equal number of male and female mice were incorporated into each treatment group. ONC212 was dissolved in sterile water fresh on the day of treatment and the mice were treated with 25 mg/kg ONC212 twice weekly via oral gavage. The mice in the vehicle group were treated with sterile water. This study was not blinded and animals with no bulk tumors were excluded from quantitative analysis.

### Immunohistochemistry

Formalin-fixed, paraffin-embedded samples were sectioned at 4 micron thickness onto Superfrost plus slides (Fisher Scientific, Waltham, MA, USA). Slides were stained with the Bond Polymer Refine Red Detection kit using a Bond Rxm automated stainer (Leica Biosystems, Wetzlar, Germany). Following a 20 min antigen retrieval in pH 6 citrate buffer (CLPP) or pH 9 Tris-EDTA buffer (SDHA) and peroxidase block in 3% hydrogen peroxide, slides were incubated with diluted primary antibody. CLPP was diluted 1:1,000 in Bond primary antibody diluent (Leica, #AR9352), and SDHA was diluted 1:10,000 in Dako antibody diluent (Agilent #S0809). Detection was performed per the standard protocol for the Bond Refine kit (Leica). A pathologist scored the IHC-stained slides, evaluating the cytoplasmic staining in the tumor cells. The results were registered as intensity; 0=negative, 1=low, 2=high, and extent; 0 - 100%, multiplying intensity and extent to derive a score from 0 to 200.

### Statistical analyses

Both metabolomics and lipidomics data were first normalized by total area normalization followed by log2 transformation and autoscaling. Hierarchical clustering heatmap was performed using relative abundance of analytes. RPPA protein expressions were similarly analyzed using heatmaps. Expression differences were also analyzed using t-tests and visualized using the Tableau Desktop software (v8.1). When comparing more than two groups, we performed an ANOVA (or the non-parametric Kruskal-Wallis test if the normal distribution requirement was not met), and if *p* < 0.05, this was followed by applying the appropriate tests for pairwise comparisons. We completed pairwise comparisons with t-test after ANOVA. We displayed the pairwise *p* values in the figures. Tumor growth figures were created with GraphPad Prism (v9). Survival analyses were performed using the Kaplan-Meier method and log-rank *p* values were calculated in R (v3.6.0) using the ‘survival’ package.

## Results

### CLPP is expressed in primary and metastatic UM

**Fig. 1:**
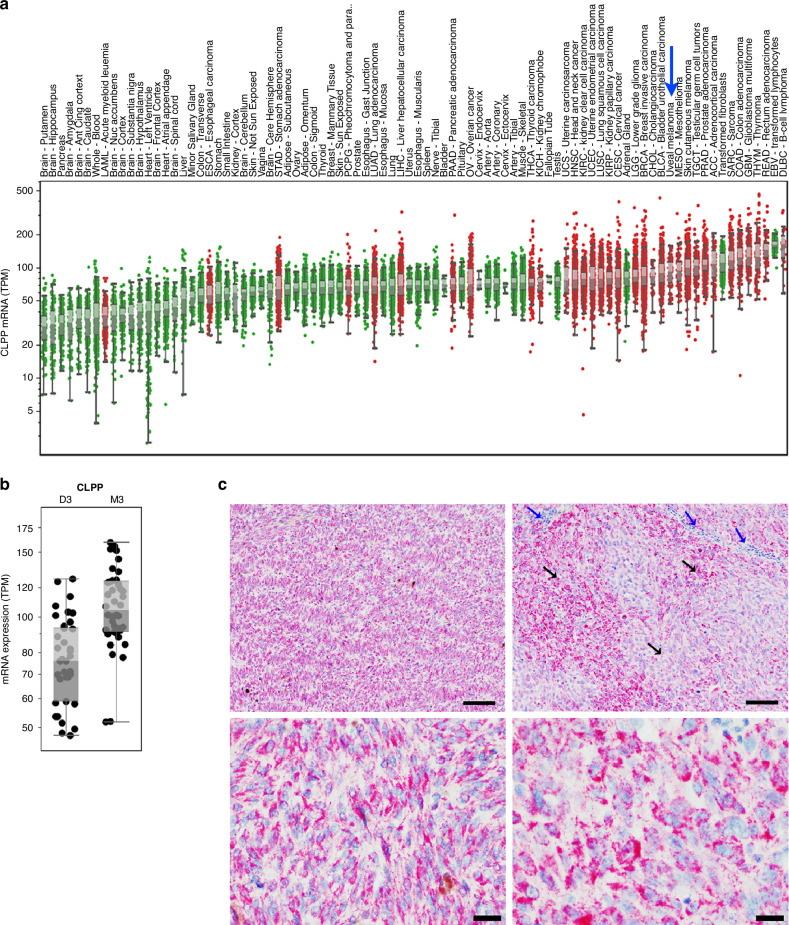
CLPP expression in primary and metastatic UM. **a** *CLPP* mRNA expression levels (transcript per million, TPM) in tumor and normal tissues were compared in 33 cancer (red dots) and corresponding normal (green dots) tissues through TCGA database analysis. The expression in UM is indicated by a blue arrow. **b** *CLPP* mRNA expression of M3 (*n* = 42) vs. D3 (*n* = 38) UM tumors from the UM TCGA. Box plots show median values and the 25th to 75th percentile range of the data (i.e., the interquartile range). **c** IHC for CLPP protein expression in mUM liver metastases from patients [10X (upper panels) and 40X (lower panels) magnifications; scale bar = 100 µm]. The blue arrows indicate stromal cells and lymphocytes, and the black arrows indicate the regions with melanoma tumors.

Mitochondrial CLPP is the known target of imipridones [20, 21]. Thus, we investigated the expression of CLPP in UM. *CLPP* gene expression was analyzed in 31 unique tumor types and their corresponding normal tissues, including primary UM tumors from The Cancer Genome Atlas (TCGA). UM tumors showed high *CLPP* gene expression amongst the evaluated cancer types (Fig. 1a). Further analysis of the TCGA UM data set revealed higher expression of *CLPP* in tumors with monosomy 3 (M3) compared to those with disomy 3 (D3) UM (*p* < 0.0001; Fig. 1b).

Immunohistochemical analysis of mUM clinical samples (*n* = 30) from various sites detected CLPP protein expression in 100% of tumor cells (representative results from two patient tumors are shown in Fig. 1c). High expression (H-score >100) of CLPP was observed in 24 (80%) samples, which included soft tissue, liver, skin, lung, small intestine, brain, and lymph node metastases. Tumor tissues showed a higher intensity of CLPP than adjacent normal tissue in all samples.

### Imipridones reduce mitochondrial OXPHOS and its effectors, and inhibit cell survival in UM cells

**Fig. 2:**
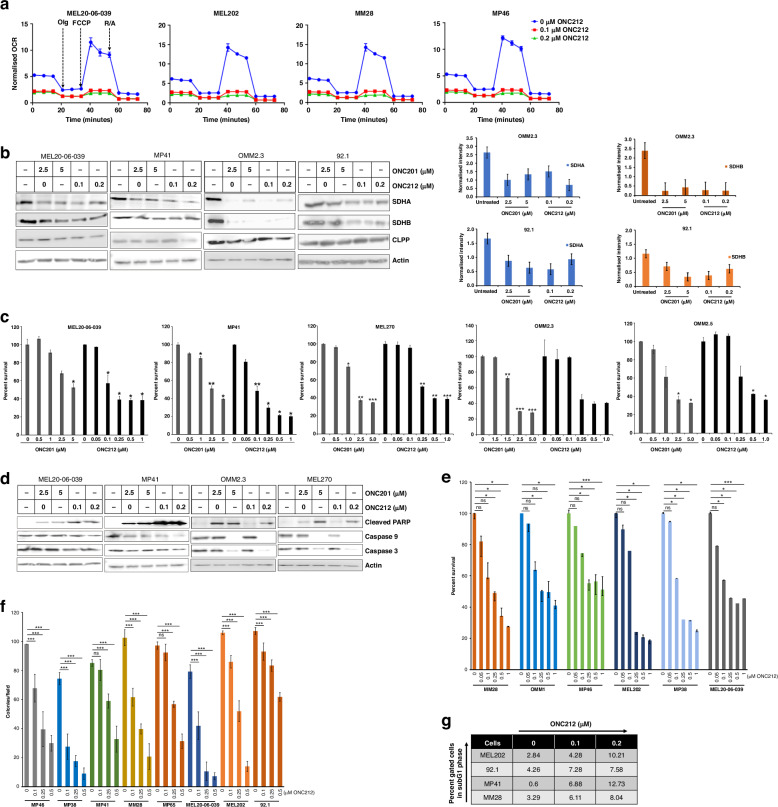
Imipridone treatment reduces OXPHOS effector proteins, inhibits cell survival, and induces apoptosis in UM cell lines. **a** Analysis of mitochondrial respiration after ONC212 treatment (0.1 and 0.2 µM) using Mito stress test Seahorse assay in UM cells (MEL20-06-039, MEL202, MM28 and MP46). The arrows represent the timepoints of addition of Oligomycin, FCCP, and Rotenone/Antimycin (R/A). The y-axis represents oxygen consumption rate (OCR, pmol/min) normalized by cell number in corresponding sample well. **b** Western blot analysis of UM cell lines (MEL20-06-039, MP41, OMM2.3, and 92.1) treated with ONC201 or ONC212 for 48 h (right panels). Quantitation of SDHA and SDHB expression levels from the western blots in 2A. Actin was used for normalization (left panels). **c** Effect of ONC201 and ONC212 on UM cell survival (in MEl20-06-039, MP41, MEL270, OMM2.3, and OMM2.5 cell lines) by MTT-based cell survival assay. Bar graphs, mean ± SD of three independent experiments. *p* values were calculated by comparison to untreated controls (*p < 0.05; **p < 0.01; ***p < 0.001). **d** Western blot analysis of cleaved PARP, intact Caspase 9, and intact Caspase 3 following treatment with ONC201 and ONC212. **e** MTT-based measurement of dose-dependent growth inhibition of UM cell lines (MM28, OMM1, MP46, MEL202, MP38, and MEL20-06-039) by ONC212. Bar graphs, mean ± SD of three independent experiments. *p* values were calculated by comparison to untreated controls (*p < 0.05; **p < 0.01; ***p < 0.001). **f** Colony formation assay: The average number of colonies observed after staining with crystal violet in ONC212 treated (0.1, 0.25 and 0.5 µM) and untreated control UM cell lines (MP46, MP38, MP41, MM28, MP65, MEL20-06-039, MEL202, and 92.1) were plotted. 5 fields/condition/cell line were counted to obtain the average number of colonies. The bar graphs represent the average number of colonies per field and are a mean ± SD of three independent experiments. *p* values were calculated by comparing untreated controls with ONC212 treatment doses (*p < 0.05; **p < 0.01; ***p < 0.001). **g** FACS analysis of PI-stained cells collected after 48 h treatment with ONC212.

ONC212 treatment markedly inhibited mitochondrial basal, maximal, and spare capacity respiration in UM cells as shown by the normalized OCR levels (Fig. 2a) in a Mito Stress Test assay. The corresponding ECAR levels are shown in Fig. S1C. ONC201 and ONC212 also reduced the levels of SDHA and SDHB in UM cell lines (MEL20-06-039, MP41, OMM2.3, and 92.1) in vitro, as detected by western blotting analysis (Fig. 2b). ONC212 inhibited the expression of SDHA and SDHB more potently than ONC201. As expected, the levels of CLPP expression were not altered with imipridone treatment.

We next evaluated the impact of ONC201 and ONC 212 on cell proliferation and survival in vitro. ONC212 was approximately tenfold more potent in inhibiting UM cell viability in MTT assays than ONC201 (Fig. 2c). Both compounds also induced apoptosis, as demonstrated by increased levels of cleaved PARP (Fig. 2d), as well as reduced levels of unprocessed caspases 3 and 9. The cleaved caspases 3 and 9 levels increased with ONC212 treatment (Fig. S2E). Similar to cell viability, ONC212 induced apoptosis more potently than ONC201.

Based on these initial results, we focused further experiments on the characterization of ONC212 in UM. ONC212 significantly inhibited UM cell growth in vitro in a dose-dependent manner in all UM cell lines tested (Fig. 2e). IC_50_ concentrations ranged from 60 to 130 nM (Fig. S1B). ONC212 also significantly inhibited colony formation in a clonogenic colony formation assay in MP46, MP38, MP41, MM28, MP65, MEL20-06-039, MEL202, and 92.1 UM cell lines (Figs. 2f and S1D). Cell cycle analysis (propidium iodide staining) revealed an increase in G0/G1 cell population with ONC212 treatment in a dose-dependent manner (Figs. 2g and S1E). All effects were observed at or near IC_50_ doses. The role of CLPP in these observed effects of ONC212 was evaluated by knocking down CLPP expression (siRNA) followed by ONC212 treatment. CLPP knockdown in MEL20-06-039 and MP46 resulted in reduced sensitivity to growth inhibition by ONC212 (Fig. S2A–C), supporting that the inhibition of cell viability by ONC212 was mediated via CLPP activation. CLPP knockdown in MEL20-06-039 also showed reduced sensitivity to ONC212-dependent OXPHOS inhibition compared to the controls (Fig. S2D).

**Fig. 3:**
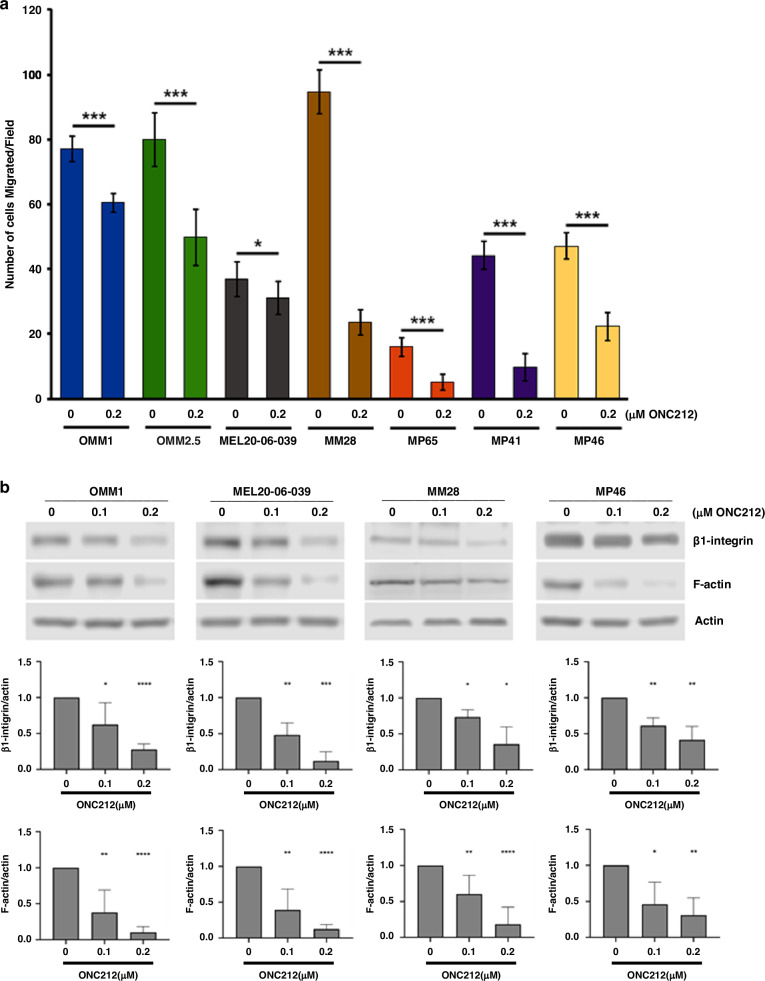
ONC212 inhibits UM cell migration. **a** In vitro cell migration assay with ONC212 treatment (0.2 µM) of UM cell lines (OMM1, OMM2.5, MEL20-06-039, MM28, MP65, MP41, and MP46). An average of five fields of cells/filter were counted under a microscope with ×40 magnification. The average number of cells counted/field were obtained in two independent experiments and the mean ± SD of these average cell counts/field were plotted as bar graphs. *p* values were calculated by comparing untreated vs. ONC212 treated cells (*p < 0.05; **p < 0.01; ***p < 0.001). **b** Western blots showing the changes in cell migration markers, β1-integrin, and F-actin upon ONC212 treatment in OMM1, MEL20-06-039, MM28 and MP46 cell lines (top panels), and corresponding quantitation of these proteins normalized with β-actin loading control (bottom panels; *p < 0.05; **p < 0.01; ***p < 0.001).

ONC212 also inhibited migration of UM cell lines, albeit to different degrees (Figs. 3a and S3). Moreover, ONC212 treatment reduced the expression of cell migration markers β1-integrin and F-actin, as shown by western blotting and quantitation of their expression (Fig. 3b).

### ONC212 treatment impacts major metabolic and cell survival pathways in UM

**Fig. 4:**
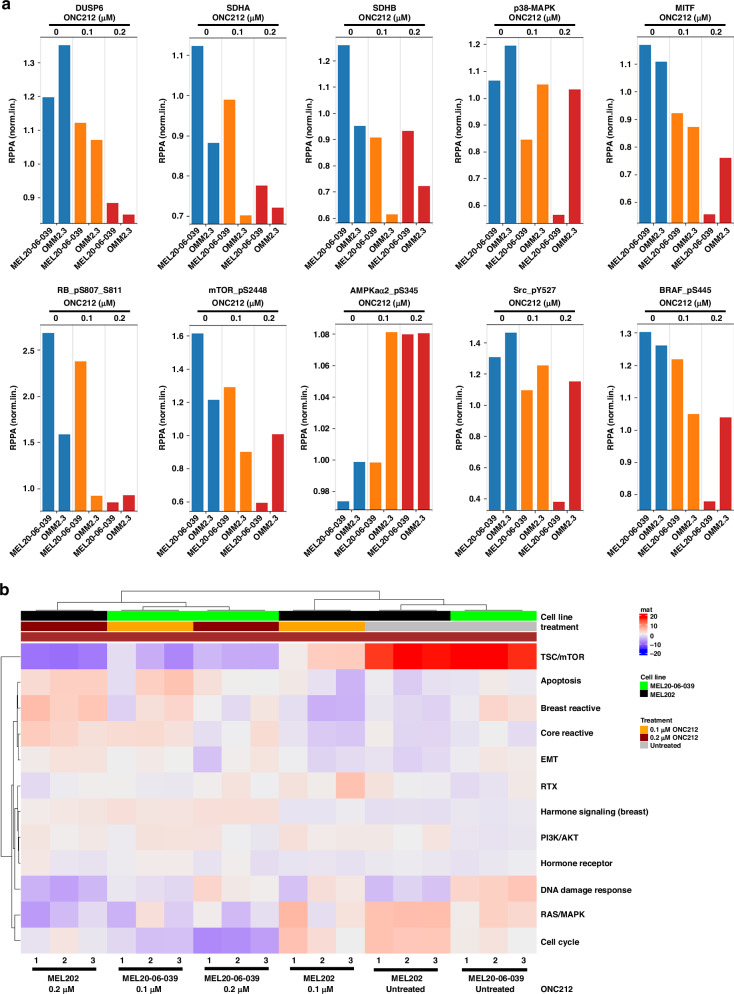
Alterations in protein profiles of UM cells with ONC212 treatment. **a** The top ten altered proteins from RPPA profiling of MEL20-06-039 and OMM2.3 UM cell lines. The RPPA expression values are displayed on the y-axis for proteins that show a significant difference (p < 0.05) compared to control sample. Untreated cells (blue bars) were compared with the cells treated for 48 h either with 0.1 µM (orange bars) or 0.2 µM (red) concentration of ONC212. **b** A heatmap of cellular signaling pathway activity scores after 48 h of ONC212 vs. vehicle treatment of UM cell lines (MEL20-06-039 and MEL202).

As OXPHOS is one of the primary energy metabolism pathways in UM cells, and a key regulator of cell survival and therapeutic resistance [38, 39], we evaluated the effect of ONC212 broadly on protein signaling pathways. UM cell lines (MEL20-06-039, MP41, MEL270, MP46, OMM2.3, and OMM2.5) were treated with 0.1 and 0.2 µM ONC212 for 48 h. RPPA proteomic analysis identified significant alterations in several important cell signaling proteins in all the cell lines tested (Figs. 4a and S4A). For clarity and space economy, representative data from two cell lines (MEL20-06-039 and OMM2.3) are shown in Fig. 4a. The normalized linear data shows a significant dose-dependent decrease in SDHA and SDHB in all the cell lines tested (p < 0.05), which indicates that ONC212 impacts OXPHOS effector levels as anticipated and is consistent with our western blotting analysis (Fig. 2a). ONC212 treatment increased levels of p85, the regulatory subunit of PI3K. ONC212 also increased AMPK and decreased mTOR activation in all UM cell lines tested (*p* < 0.05), suggesting ONC212 induces metabolic stress. Glucose metabolism modulators such as DUSP6 and AKT2 were also decreased with ONC212 treatment (*p* < 0.05). Retinoblastoma (Rb) protein phosphorylation at S807-811 was decreased (*p* < 0.05). This Rb phosphorylation regulates interaction of Rb with pro-apoptotic BAX protein [40]. These results suggest that ONC212 treatment affects major metabolic and apoptotic proteins in UM cells.

We performed pathway analysis on RPPA data for MEL202 (D3) and MEL20-06-039 (M3) UM cell lines after treatment with ONC212 (0.1 and 0.2 µM) in triplicate for 24 and 48 h (Figs. 4b and S4B). ONC212 treatment downregulated mTOR signaling (≥20-fold) and MAPK pathway signaling (≥10-fold) and upregulated apoptotic pathways (~10-fold). Additionally, we observed a significant difference in the expression of proteins involved in the DNA damage response between the D3 and M3 cell lines at baseline. Therefore, the RPPA data indicates that ONC212 impacts UM cellular metabolism and survival.

### ONC212 inhibits protein metabolism and lipid biosynthesis in UM

**Fig. 5:**
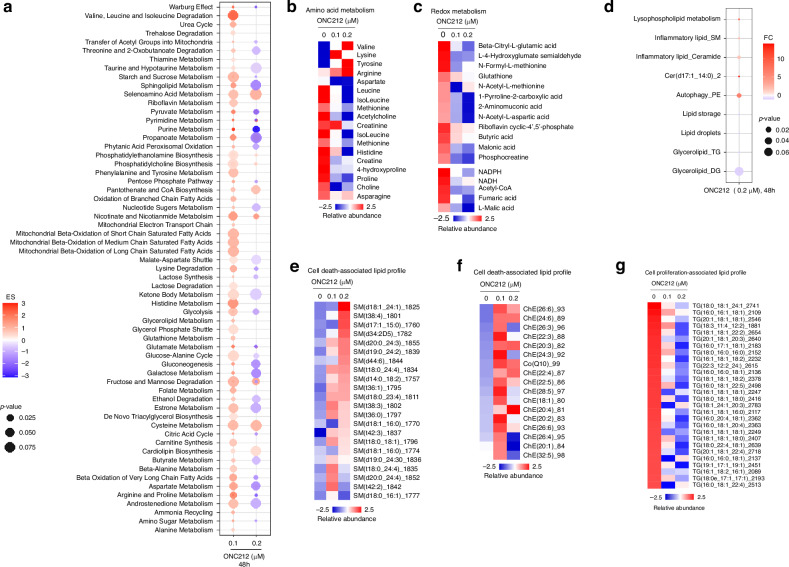
Alterations in metabolomic and lipidomic profiles of UM cells with ONC212 treatment. **a–g** Mass spectrometry-based global metabolomics and lipidomics of UM cell line MEL20-06-039, treated with 0.1 and 0.2 µM of ONC212 for 48 h. **a** Pathway analysis and trends from significant changes observed in the metabolic profile. Heatmap showing changes in essential amino acids (**b**), and changes in metabolites involved in redox metabolism (**c**). **d** Pathway analysis and trends using lipidomics profiling showing significant changes upon ONC212 treatment. Heatmaps of lipid profiles analysis in Sphingomyelin (SM) (**e**), in inflammatory lipid signature such as ceramide, cholesterol ester, and CoQ (**f**), and in triglyceride signature (TG) (**g**).

Our prior work showed that M3UM relies on high OXPHOS and high expression of mitochondrial OXPHOS effectors for survival [16]. Since ONC212 treatment inhibited UM cell survival (Fig. 2), we investigated whether this treatment alters the metabolism of UM cells. MEL20-06-039 cells were treated with ONC212 (0.1 and 0.2 µM), and the metabolites were extracted for high-resolution mass spectrometry-based global metabolomics and lipidomics profiling. Global pathway analysis showed that ONC212 treatment significantly reduces energy metabolism pathways in a dose-dependent manner (Figs. 5a and S5A). ONC212 treatment diminished ROS regulating metabolites involved in glutathione, aspartate-glutamate, and NADH metabolism (Fig. 5a). ONC212 also reduced the levels of the essential amino acids- with the exception of an increase in levels of Valine, Lysine, Tyrosine, and Arginine (Fig. 5b), which may be involved as intermediates in perturbation of protein metabolism upon ONC212 treatment. Metabolites involved in redox metabolism were also markedly reduced with ONC212 treatment (Fig. 5c), suggesting that ONC212 affects amino acid and redox metabolism. Moreover, ONC212 decreased metabolites involved in macromolecule metabolism such as nucleotide biosynthesis and amino sugar metabolism (Fig. S5B). In contrast, ONC212 treatment resulted in enrichment of a set of signature metabolites including itaconic acid, lactic acid, and TCA cycle intermediates (Fig. S5C). These metabolites and their downstream networks are often involved in cell death and inflammation in response to drug treatment [41].

Pathway enrichment analysis revealed differential enrichment of lipid ontologies by ONC212 (Fig. 5d). ONC212 increased levels of cell-death and inflammatory lipids like sphingomyelin (SM) (Fig. 5e), cholesterol ester (ChE), and coenzyme Q (CoQ) (Fig. 5f). In contrast, lipids such as triglycerides (TG), which is essential for cell proliferation, decreased (Fig. 5g). The detailed heatmaps of the global lipidomics profiling are provided in Fig. S5D–F. Collectively, ONC212 alters the UM cell lipidome by elevating lipids linked to inflammation and cell death while suppressing proliferative lipids [42].

### ONC212 inhibits UM liver metastasis growth and improves survival in vivo

**Fig. 6:**
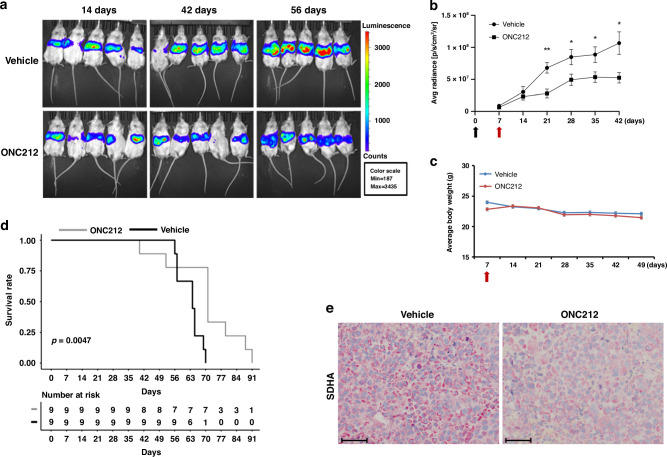
ONC212 reduces tumor burden and improves survival in UM liver metastasis model. **a** Representative images of weekly bioluminescence scans of mice with liver metastases of UM 92.1 treated with vehicle (control) or ONC212 (25 mg/kg). **b** Tumor growth curves plotted from bioluminescence scans post-tumor initiation. The black arrow represents the splenic injection, and the red arrow indicates the beginning of treatment. **c** Mouse body weight with vehicle and ONC212 treatment; red arrow indicates beginning of treatment. **d** Kaplan-Meier plots of 92.1 model with ONC212 treatment compared to vehicle treated controls; *n* = 10 per treatment group; 92.1 vehicle vs. ONC212 *p* = 0.0047; Hazard ratio from Cox proportional hazards model: 7.80 [95% confidence interval: 1.573 to 38.66]. **e** Representative images (scale bar = 100 µm) of SDHA immunohistochemical staining in vehicle and ONC212-treated 92.1 tumors in liver.

We utilized an orthotopic model of UM liver metastasis to study the effects of ONC212 in a clinically relevant model. Luciferase tagged UM cells (92.1, MEL20-06-039, MP41 and MM28) were injected into the spleen of NSG mice, followed by splenectomy [36]. After confirming tumor growth in the liver, mice were randomized and treated with 25 mg/kg ONC212 or vehicle twice weekly. Tumor burden was monitored through weekly IVIS scans (Figs. 6a and S6A).

Treatment with ONC212 significantly reduced liver tumor burden of hepatic xenografts of 92.1 and MEL20-06-039 (Figs. 6b and S6B) and improved overall survival (Figs. 6d and S6D). Of note, with prolonged ONC212 treatment (9-10 wks) we observed tumor recurrence, indicating the development of ONC212 resistance (Figs. 6a and S6A). No significant changes in mouse body weight were observed during ONC212 treatment (Figs. 6c and S6C). ONC212 did not improve survival in mice with hepatic xenografts of MP41 and MM28 (Fig. S7). Mouse livers were excised at the end of the survival analysis for further study. IHC analysis showed a reduction in SDHA protein in the ONC212-treated vs. vehicle treated 92.1 UM xenografts (Figs. 6e and S6E). Taken together, our data indicates a beneficial treatment effect of ONC212 in 2 of 4 models of UM liver metastasis.

## Discussion

There is a critical need for new strategies for UM. Previous studies by our group, subsequently and independently validated by others, identified elevated mitochondrial OXPHOS and high expression of OXPHOS effector proteins in mUM [16, 20, 43]. Upon targeting OXPHOS directly using IACS10759 in UM cell lines, we observed limited reduction of cell viability. This poor response was due to elevated expression of OXPHOS effectors [16]. Moreover, a phase 1 clinical study with IACS10759 resulted in unacceptable toxicities (NCT 03291938) [18]. Therefore, there was a necessity for a safe and effective alternate strategy to target the OXPHOS-dependency of UM. In this study, we show that the imipridones ONC201 and ONC212, which inhibit OXPHOS indirectly by activating mitochondrial CLPP, suppress basal, maximal, and reserve mitochondrial respiration, reduce OXPHOS effectors; inhibit UM cell survival and migration; and induce cell death in vitro. ONC212 treatment also affected multiple signaling and metabolic pathways in UM cells. Further, ONC212 significantly reduced tumor burden and improved survival in two of four in vivo models of UM liver metastasis. Together these results support that imipridones may be a novel therapeutic strategy for mUM.

This is the first attempt, to our knowledge, to inhibit UM using imipridones. Mitochondrial CLPP regulates OXPHOS through proteolysis of effector proteins such as SDHA and SDHB [44]. Imipridones indirectly regulate OXPHOS levels through modulation of CLPP activity. ONC201 has been shown to suppress cellular OXPHOS in medulloblastoma cells [45]. Though CLPP has been identified as the target of ONC212, the mechanism of cancer cell death induced by ONC212 is not well-understood [46]. To identify mechanism(s) of ONC212 action in UM cells, we analyzed multiomics data, including proteomic, metabolomic and lipidomic profiles. These analyses highlighted the activation of apoptotic pathways and induction of metabolic stress by ONC212 treatment. In pancreatic cancer, ONC212 has been shown to collapse mitochondrial function [46]. We observed that the mitochondrial proteins SDHA and SDHB were significantly reduced by ONC212, consistent with CLPP activation. Additionally, we observed alterations to AMPK and mTOR levels. AMPK and mTOR are primary regulators of cellular metabolism, as they sense and respond to metabolic environment and oxidative stress. Activated AMPK has also been shown to inhibit mTORC1 signaling [47]. Our observation of higher AMPK phosphorylation and lower mTOR with ONC212 treatment suggests an induction of metabolic stress in these cells.

Since our proteomics data suggests that ONC212 reduces the levels of OXPHOS effectors and induces metabolic stress in UM cells, we further dissected the metabolic effects of ONC212 treatment in UM cells. Global metabolomics and lipidomics analysis identified downregulation of ROS neutralizing entities such as glutathione and polyamines by ONC212, implicating ROS upregulation in cell death with this treatment. There was also a significant impairment of protein biosynthesis, which may further contribute to ONC212-induced cell death. Our metabolic analysis also shows a reduction in Warburg effect. An investigation of the lipid profiles show an accumulation of inflammatory lipids that are known to induce cell death [48, 49]. Moreover, a reduction in triglycerides signify an inhibition of the cell proliferation pathways with ONC212 treatment.

Most importantly, our studies show that ONC212 reduced UM tumor growth in vivo in two different orthotopic mouse models for mUM liver metastasis. The liver is the most frequent site of UM metastasis, and liver involvement has been associated with poor outcomes with other therapies, including immune checkpoint inhibitors. Thus, the observed results with single-agent ONC212 in this aggressive, clinically relevant model are promising and clinically relevant. However, there is clearly a need to build upon this initial demonstration of activity. The heterogeneous outcomes observed in the 4 UM models assessed, along with the eventual development of resistance in the models that demonstrated initial benefit, provide a foundation for further characterization of the determinants of the efficacy of ONC212 for mUM- and hopefully the development of even more effective combinatorial strategies.

## Supplementary information

Supplemental figures and legends

## Data Availability

All relevant data are available from the corresponding author upon request.
