# Supplementary material for: Imipridones inhibit tumor growth and improve survival in an orthotopic liver metastasis mouse model of human uveal melanoma
Source: Br J Cancer. 2024 Oct 11;131(11):1846–57. doi: 10.1038/s41416-024-02866-6 (PMC11589887; doi:10.1038/s41416-024-02866-6)
Supplement: Supplementary file 1 — Supplemental figures and legends [file 41416_2024_2866_MOESM1_ESM.pdf]

Supplementary Figure 1

A

Cell Line	Chromosome 3 Status
MM28	M3
OMM1	D3
MP46	M3
MEL202	D3
MP38	M3
MEL20-06-039	M3
OMM2.5	D3
92.1	D3
MP41	D3
MEL270	D3
OMM2.3	D3
MP65	M3

B

Cell Line	ONC212 IC_{50} (nM)
MM28	116
OMM1	82.4
MP46	92.8
MEL202	130
MP38	91.3
MEL20-06-039	62.1

C

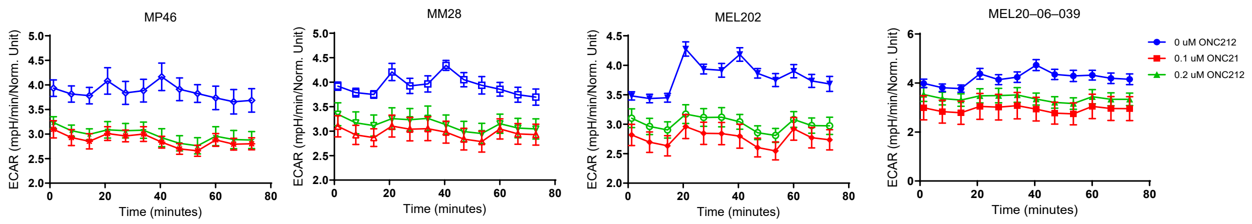

D

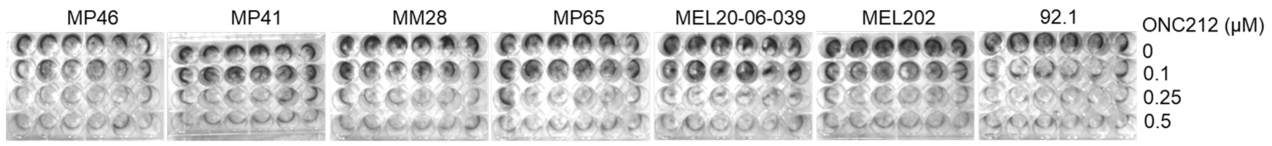

E

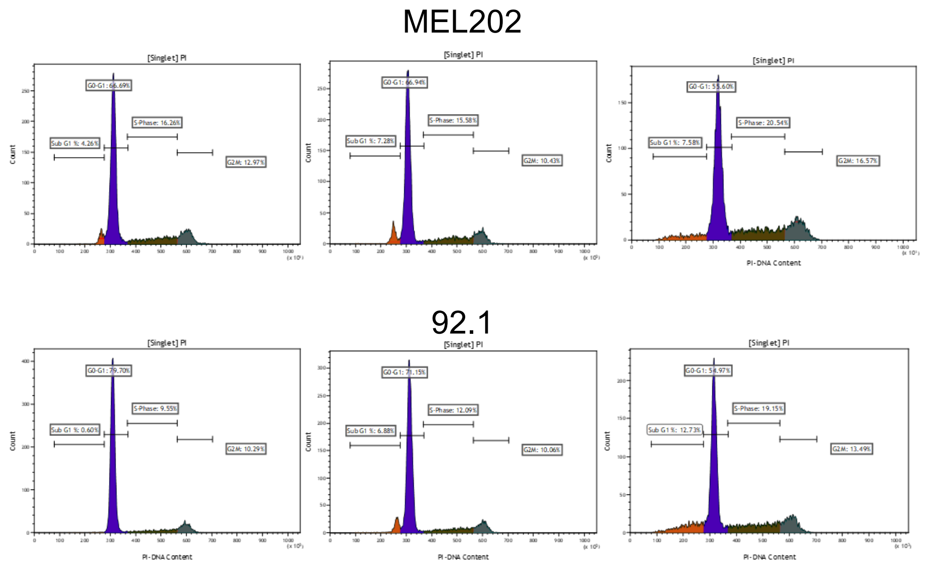

UM cell lines used in this study and the effect of ONC212 on UM cell survival and OXPHOS **(A)** Chromosome 3 status of UM cell lines. **(B)** IC_{50} values of ONC212. **(C)** Mito stress test Seahorse assay. **(D)** Colony formation assay. **(E)** Representative FACS histograms of G0/G1 phase with ONC212 treatment.

Supplementary Figure 2

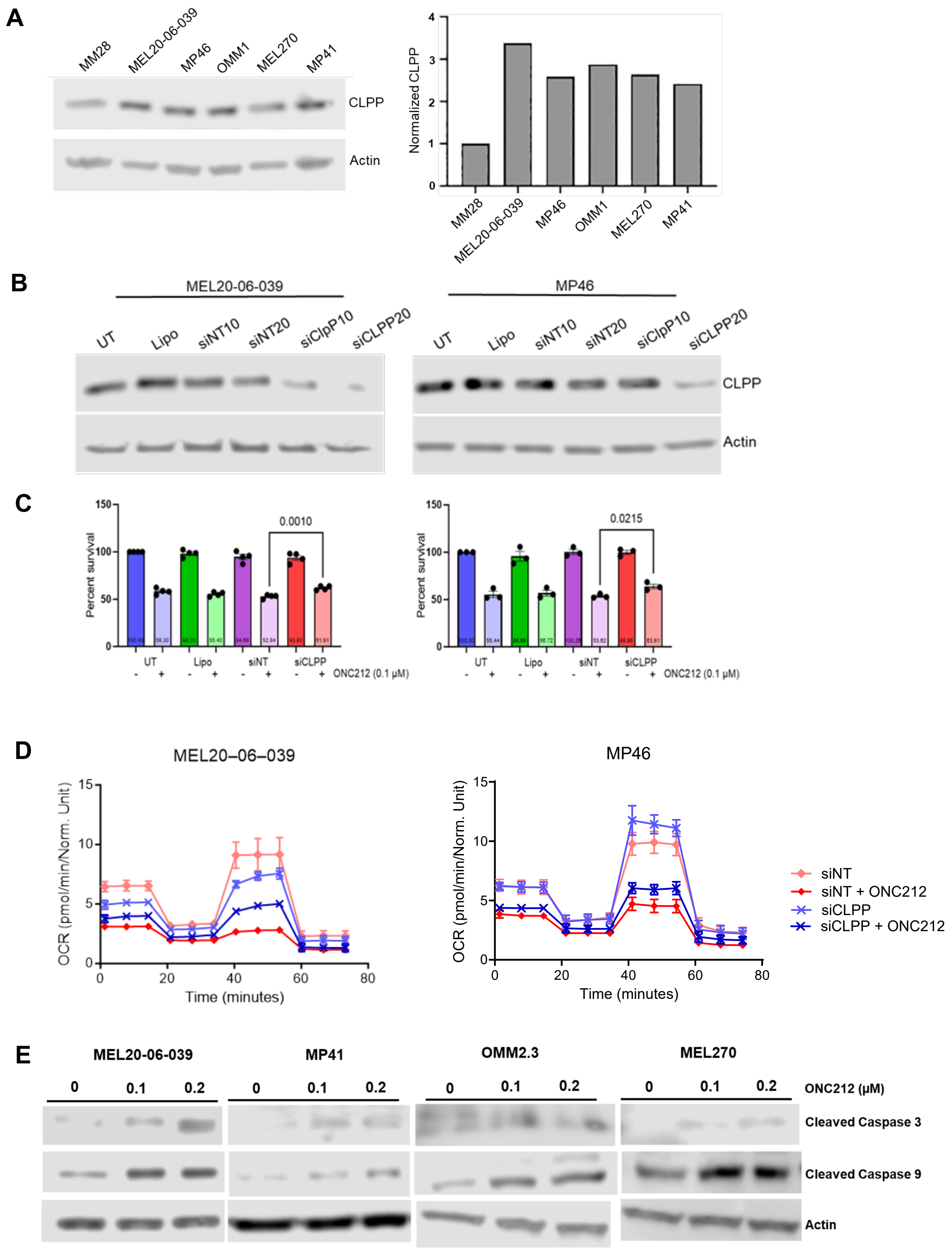

CLPP as ONC212 target in UM cells. **(A)** Western blot (left) showing the levels of CLPP expression in different UM cell lines, and bar graphs (right) showing the corresponding normalized CLPP expression. **(B)** Western blots showing CLPP knockdown using siRNA (10 and 20 nM siCLPP). The controls were Untreated (UT), Lipofectamine-treated (Lipo), Non-targeting siRNA-treated (NT) cells. **(C)** Cell viability assay post-*CLPP* knockdown and ONC212 treatment (0.1 μM). **(D)** Mito stress test assay post-*CLPP* knockdown (10 nM siRNA) and ONC212 treatment (0.1 μM) in MEL20-06-039 and MP46 cells. **(E)** Western blots showing the levels of cleaved caspase 3 and Caspase 9 post ONC212 treatment in UM cell lines.

Supplementary Figure 3

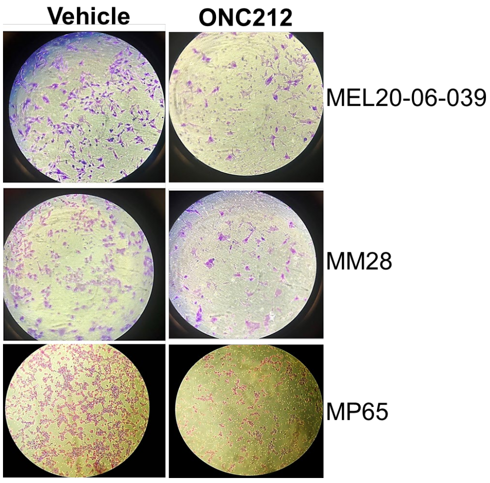

ONC212 inhibits UM cell migration Representative brightfield images (10X) of cell migration assay in UM cell lines, MEL20-06-039, MM28 and MP65, treated with vehicle or 0.2 μM ONC212.

Supplementary Figure 4

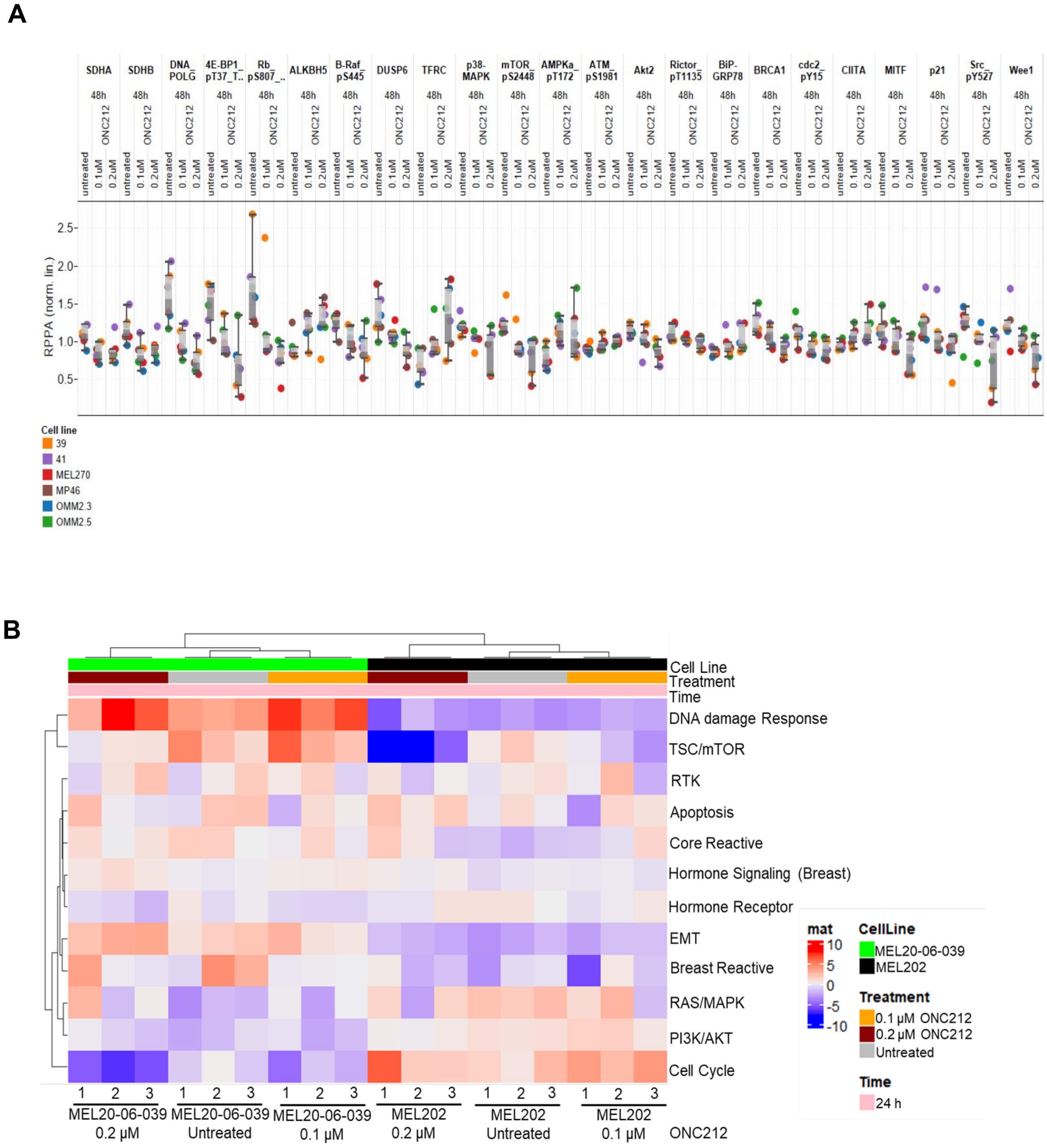

ONC212 alters global proteomics of UM cells. (A) RPPA protein profiling of 6 UM cell lines (MEL20-06-039, MP41, MEL270, MP46, OMM2.3, and OMM2.5), represented by heatmaps. Cells were treated for 48 h with either 0.1 or 0.2 µM ONC212. **(B)** 24 h RPPA data set supplementing Figure 4B.

Supplementary Figure 5

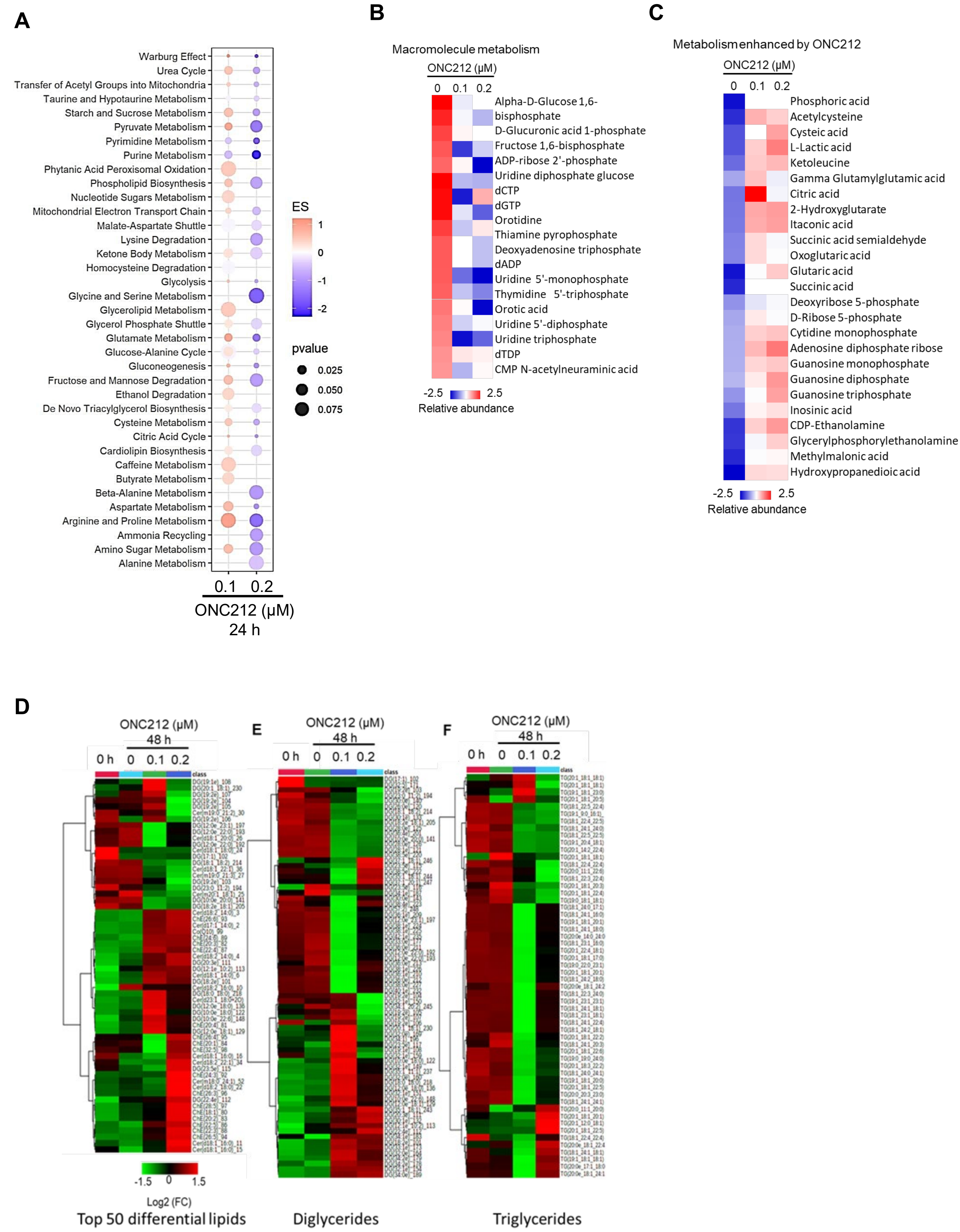

ONC212 alters global metabolomic and lipidomics profiles of UM cells with 24 h treatment. (A) Pathway analysis and trends from significant changes in the metabolic profiles. **(B)** Heatmaps of macromolecule metabolism and **(C)** metabolites enhanced by treatment. **(D-F)** Heatmaps of lipid profiles.

Supplementary Figure 6

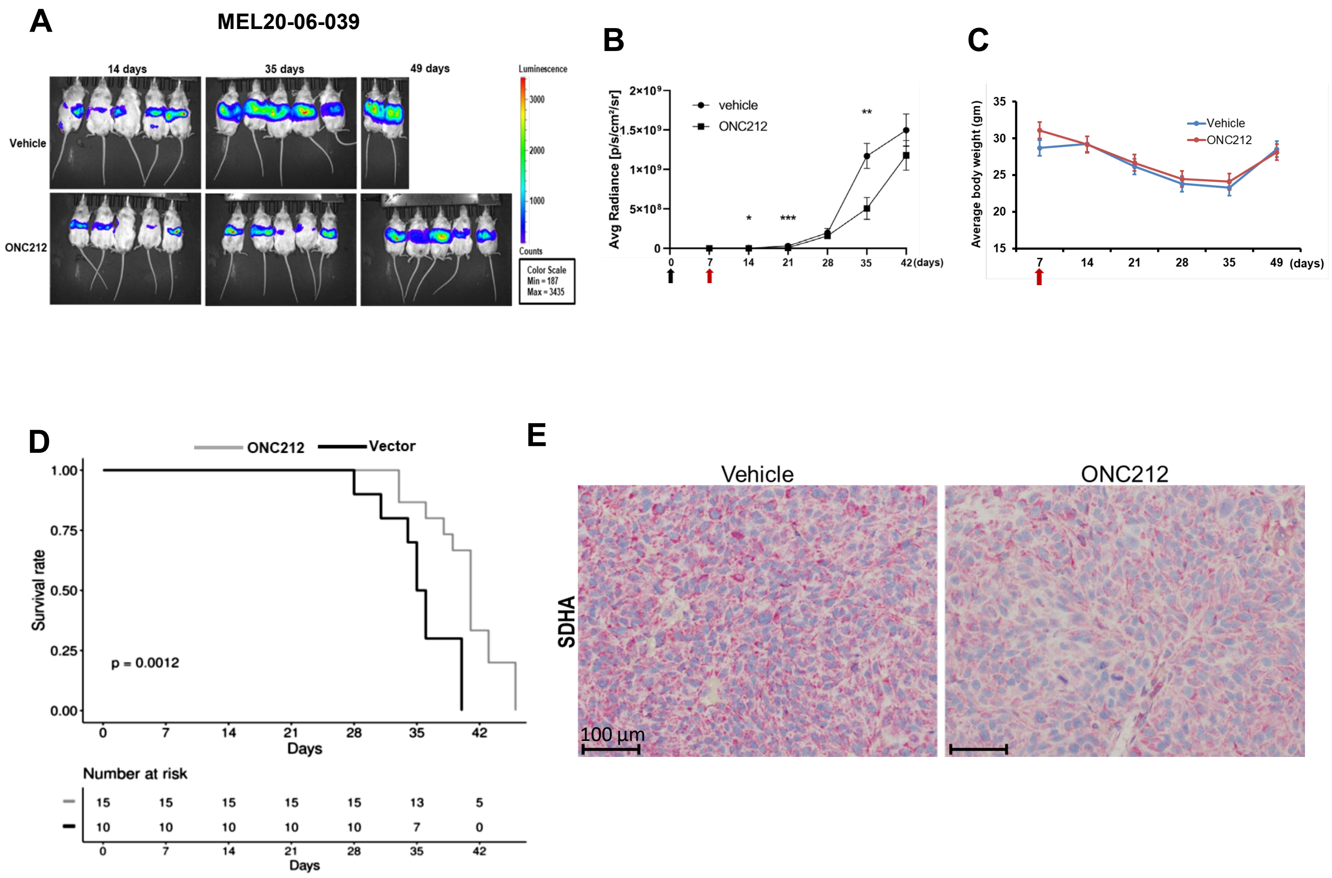

ONC212 reduces tumor burden and improves survival in MEL20-06-039 preclinical model. (A) Bioluminescence scans. **(B)** Tumor growth curves. The black and red arrows represent splenic injection and beginning of treatment. **(C)** Mouse body weight plots. **(D)** Kaplan-Meier plots; Hazard ratio: 10.60 [95% CI: 2.205 to 50.96] **(E)** SDHA IHC.

Supplementary Figure 7

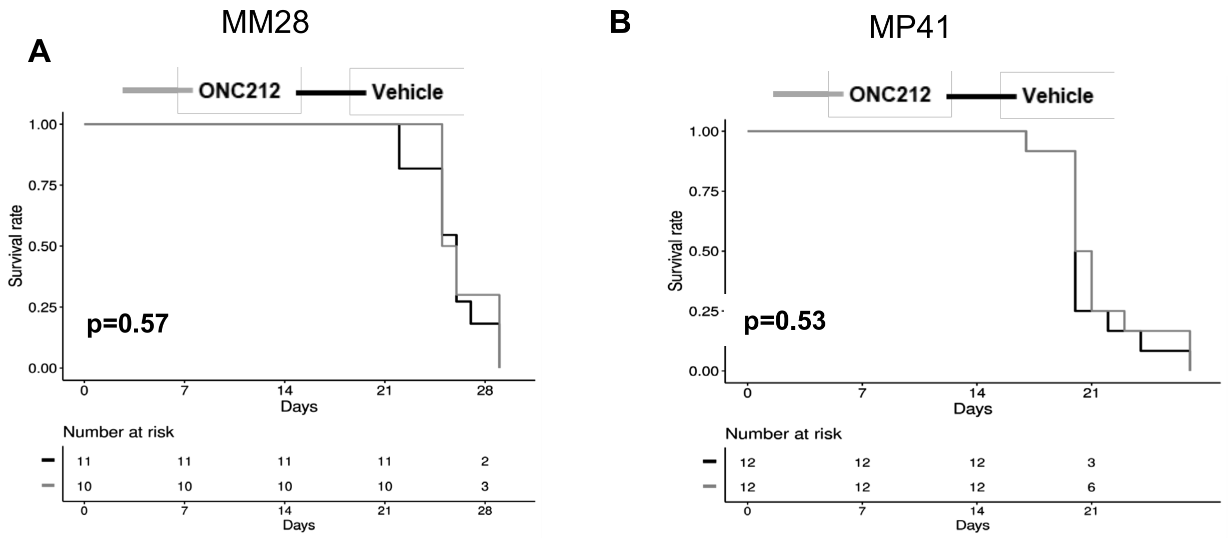

ONC212 does not improve survival in MM28 and MP46 preclinical models. Kaplan-Meier plots and Hazard Ratios of **(A)** MM28 and **(B)** MP41 models with ONC212 treatment ; n = 10 per treatment group; vehicle vs. ONC212 treatment are p = 0.57 and p = 0.53, respectively.

Supplementary Figure 8

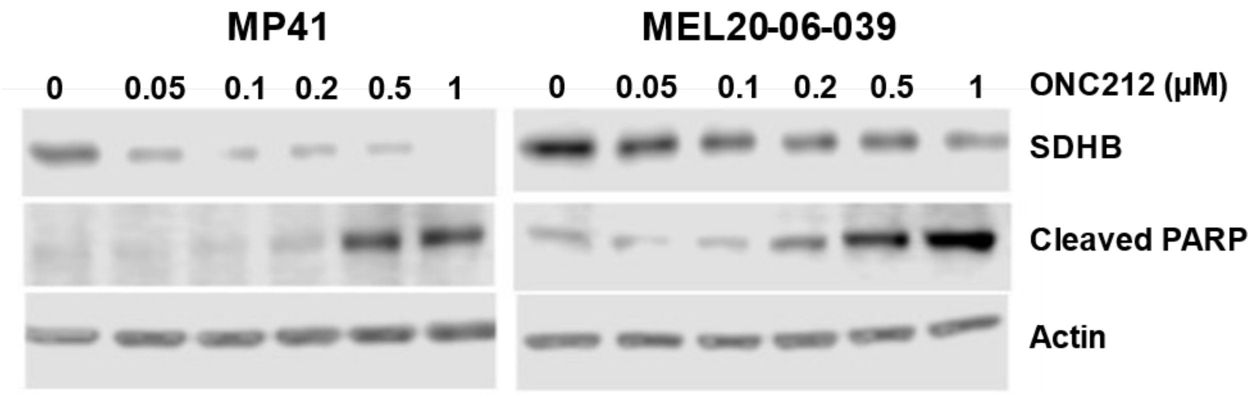

Dose dependent effect of ONC212 on metabolic and apoptotic markers. Western blot analyses of SDHB (metabolic marker) and cleaved PARP (apoptotic marker) levels in MP41 and MEL20-06-039 cells treated with increasing doses of ONC212.
